# Novel copy number variation of *POMGNT1* associated with muscle-eye-brain disease detected by next-generation sequencing

**DOI:** 10.1038/s41598-017-07349-8

**Published:** 2017-08-01

**Authors:** Xiaona Fu, Haipo Yang, Hui Jiao, Shuo Wang, Aijie Liu, Xiaoqing Li, Jiangxi Xiao, Yanling Yang, Xiru Wu, Hui Xiong

**Affiliations:** 1 Department of Pediatrics, Peking University First Hospital, Beijing, 100034 China; 2 Department of child ophthalmology, Peking University First Hospital, Beijing, 100034 China; 3 Department of Radiology, Peking University First Hospital, Beijing, 100034 China

## Abstract

The protein O-mannose beta-1,2-N-acetylglucosaminyltransferase 1 (POMGNT1) gene is one of 18 genes involved in the pathogenesis of α-dystroglycanopathies(α-DGPs) such as muscle–eye–brain disease (MEB). Our study aimed to retrospectively analyze and characterize the clinical and genetic features of three MEB patients with *POMGNT1* mutations. One female and two male patients from three unrelated families were diagnosed with MEB, manifesting hypotonia at birth, mental retardation, structural brain defects, and ocular malformations. The novel missense mutations c.296 T > C and c.794 G > C were revealed in patient 2 and patient 3 respectively by next-generation sequencing (NGS). Further NGS data analysis revealed that all three patients had the same novel copy number variations (CNV) g.6668-8257del, which was homozygous in patient 1 and heterozygous in patients 2 and 3. By long-range polymerase chain reaction (PCR) and Sanger sequencing, it was shown that the two breakpoints of the CNV localized to two *Alu*Y elements and displayed 42-bp of microhomology. The CNV was confirmed as a founder mutation by haplotype analysis. Our study indicated that NGS is a clinically useful method of detecting α-DGPs genes -related CNV, and the CNV is likely to be caused by *Alu-Alu* recombination or from a single ancestor bearing the deletion chromosome.

## Introduction

The protein O-mannose beta-1,2-N-acetylglucosaminyltransferase 1 (POMGNT1) is one of the pathogenic genes of α-dystroglycanopathies (α-DGPs) which are caused by glycosylation defects of α-dystroglycan (α-DG)^1, 2^. α-DGPs are a heterogeneous group of muscular dystrophies with autosomal recessive inheritance^3^, and α-DGPs have been renamed muscular dystrophy-dystroglycanopathies (MDDG). Current known MDDG types are from A (most severe) to C (least severe). Until now, 18 genes had been discovered to be pathogenic for α-DGPs, including the following: *POMT1*(OMIM #607423), *POMT2*(OMIM #607439), *POMGNT1*(OMIM #606822), *FKTN*(OMIM #607440), *FKRP*(OMIM #606596), *LARGE*(OMIM #603590), *GTDC2/POMGNT2*(OMIM #614828), *B3GALNT2*(OMIM #610194), *B3GNT1*(OMIM #605517), *SGK196/POMK*(OMIM #615247), *TMEM5*(OMIM #605862), *GMPPB*(OMIM #615320), *DPM1*(OMIM #603503), *DPM2*(OMIM #603564), *DPM3*(OMIM #605951), *DOLK*(OMIM #610746), *ISPD*(OMIM #614631), and *DAG1*(OMIM #128239)^4^. Mutations in the *POMGNT1* are responsible for several forms of α-DGPs such as Walker–Warburg syndrome (WWS, OMIM #253280, renamed MDDGA3)^5^, muscle–eye–brain disease (MEB, OMIM #253280, renamed MDDGA3)^1^, congenital muscular dystrophy with mental retardation (OMIM #613151, renamed MDDGB3)^6^, and limb girdle muscular dystrophy type 2 O (LGMD2O, OMIM #613157, renamed MDDGC3)^7^. Additionally, it has been reported that mutations in the *POMGNT1* can cause retinitis pigmentosa-76 (RP76, OMIM#606822)^8, 9^. With the broad clinical application of next-generation sequencing (NGS), more and more patients with different phenotypes have been diagnosed with α-DGPs^10^. However, molecular diagnosis by NGS is not always successful because the disease may be caused by as of yet undiscovered genes or sequencing of the known genes might be incomplete. To resolve the latter problem, we analyze the number of sequence reads of patients along with control samples from NGS, and we report here a novel copy number variation (CNV) of the *POMGNT1* in three MEB patients from three unrelated families. To our knowledge, this is the first study to describe NGS as a tool for identifying CNV of α-DGPs genes. Furthermore, two possible causes of this CNV were discussed as *Alu-Alu* recombination and founder mutation.

## Results

### Phenotypic features of patients

#### Patient 1

**Figure 1:**
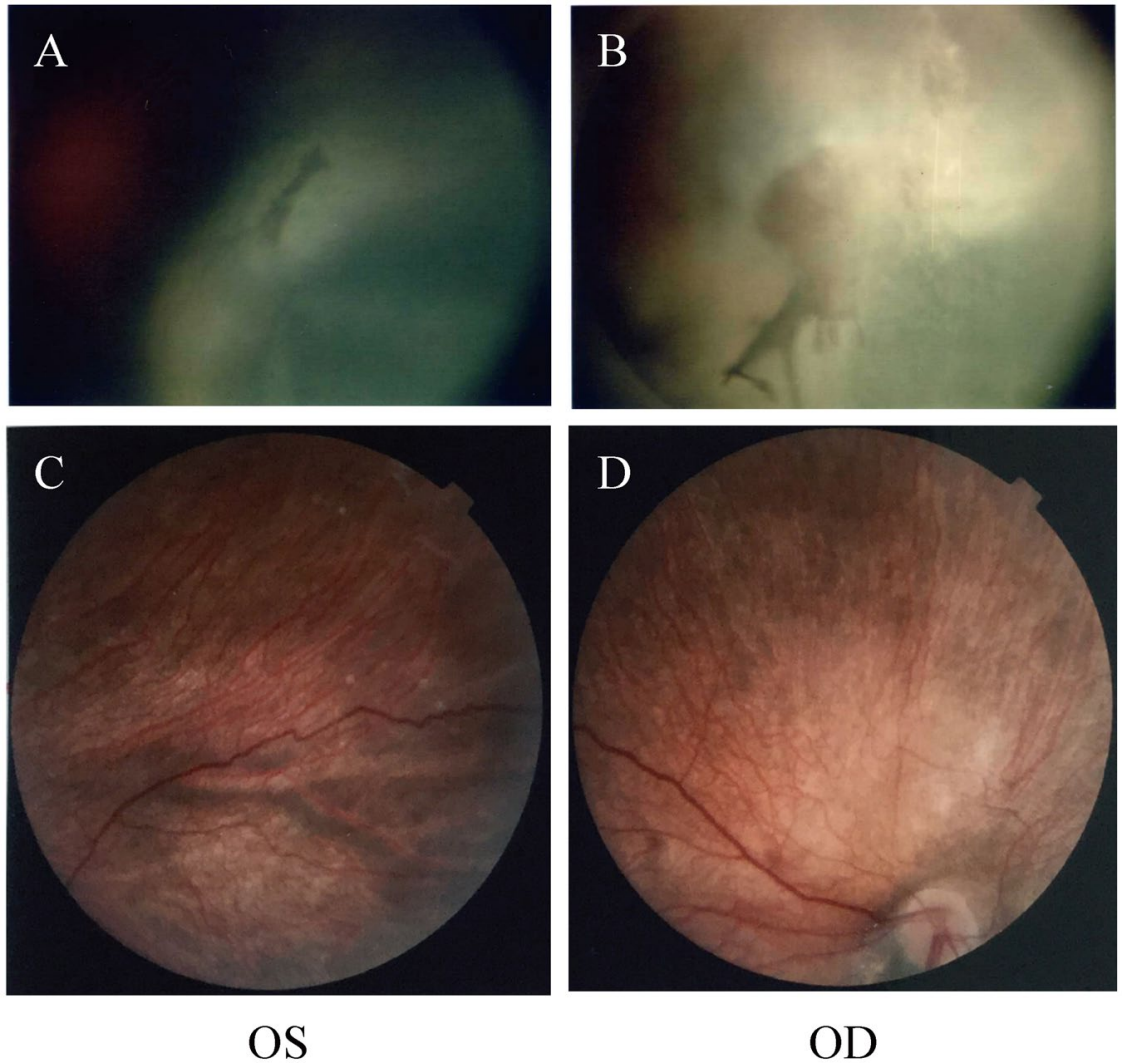
Fundus photography of patient 1 and patient 3. Fundus photography in patient 1 (**A** and **B**) showing right (OD) cataract, left (OS) optic nerve atrophy, and left retinal dysplasia. Fundus photography in patient 3 (**C** and **D**) showing retinal and choroidal pigment epithelium atrophy and optic nerve atrophy.

**Figure 2:**
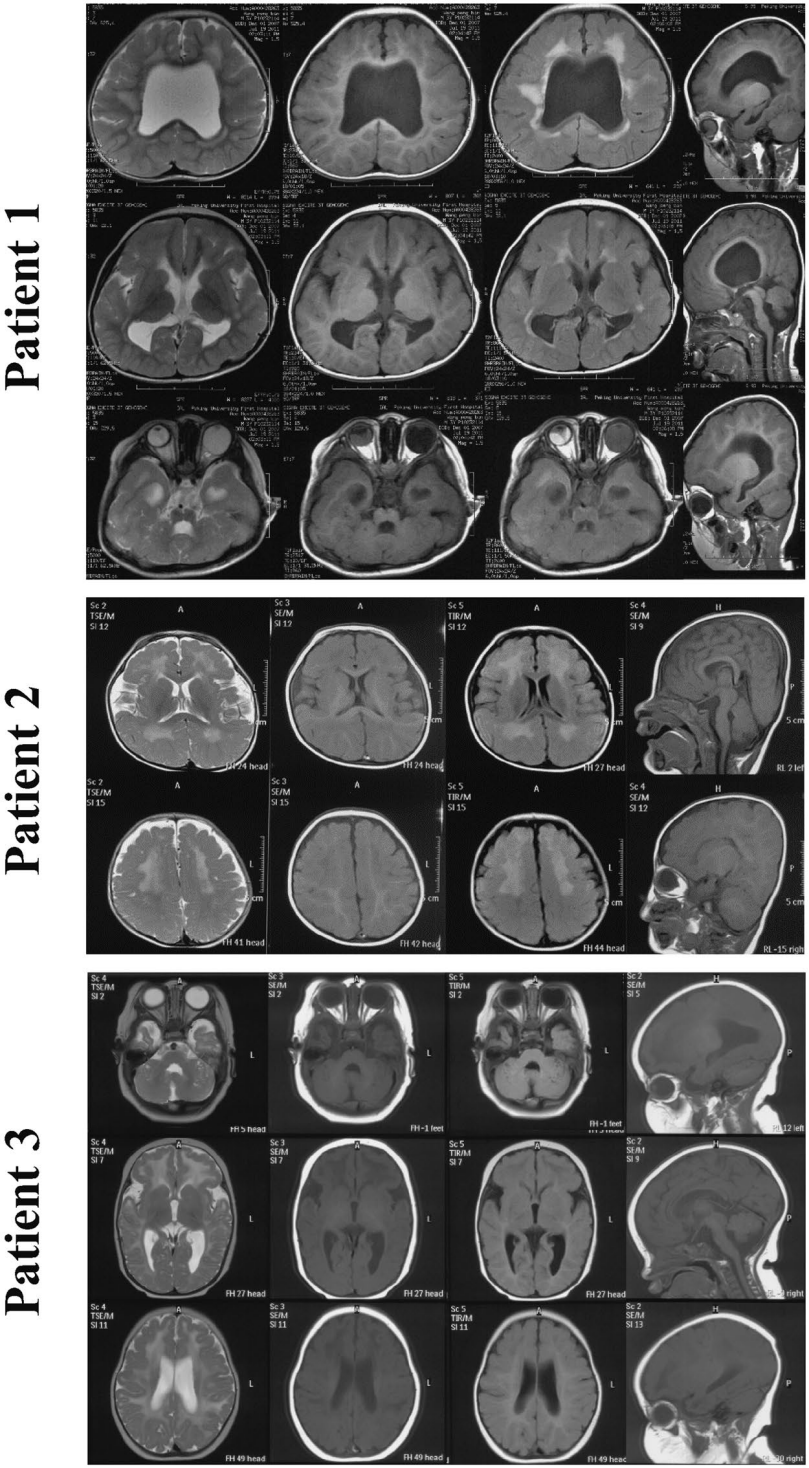
Brain MRI of all three patients. Patient 1 underwent a brain MRI at 3 years and 6 months of age, and showed micropolygyria, septum pellucidum absence, corpus callosum dysplasia, cerebellum hypoplasia, and cerebellar cysts with abnormal white matter signals in T1W and T2W. A brain MRI of patient 1 also showed right microphthalmus, amotio retinae, and left eye irregular shape. Patient 2 underwent a brain MRI at 8 months of age and demonstrated frontal lobe micropolygyria, increased white matter signals in T2W and fluid attenuated inversion recovery (FLAIR), and dysplasia of the brainstem and cerebellum. Patient 3 underwent a brain MRI at 4 months of age, and showed frontal lobe micropolygyria, abnormal white-matter signals in the bilateral frontal region, dysplasia of the brainstem and cerebellum, and cerebellar cysts with abnormal white matter signals in T1W, T2W and FLAIR.

Patient 1 was an 8-year-5-month-old male, who displayed significant motor and cognitive developmental delay with mental retardation. He was the second child, born at full-term to a healthy woman by normal spontaneous vaginal delivery, with a birth weight of 2.5 kg. He had an unaffected older sister. His parents were consanguineous Han from Gansu Province. The mother’s history included two spontaneous abortions. 24 weeks into the mother’s fifth pregnancy, fetal hydrocephalus was detected and the parents selected termination of pregnancy (Pedigree shown in Supplementary Figure S1). Patient 1 had never achieved head control or the ability to sit unsupported. He could turn over by himself when he was 5 years old. He could neither speak nor communicate with others. Upon physical examination, he showed a myopathic face, a high-arched palate, nystagmus, generalized muscle weakness, and marked hypotonia. Deep tendon reflexes were absent. Both knee and ankle joint contractures were present. Right Babinski sign was positive, and left Babinski was probable positive. Ophthalmic examination demonstrated microphthalmia, microcornea, right cataract, left severe myopia, left optic nerve atrophy, and left retinal dysplasia (Fig. 1). The serum CK level was 1514 U/L. The brain magnetic resonance imaging (MRI) showed micropolygyria, septum pellucidum absence, corpus callosum dysplasia, cerebellum hypoplasia, and cerebellar cysts. The brain MRI also showed right microphthalmus, amotio retinae, and irregular shape of the left eye (Fig. 2).

#### Patient 2

Patient 2, a 3-year-1-month-old male with motor and cognitive delay, was the first child, born at full-term to a healthy woman, with a birth weight of 2.8 kg. His parents were non-consanguineous Han from Anhui Province (Pedigree shown in Supplementary Figure S1). He achieved head control at age 1, could turn over at age 1 and a half, and could sit unsupported at age 2. He could not speak, but could understand easy instructions. Upon physical examination, he showed nystagmus, hypertonia of the extremities, trunk hypotonia, and ankle joint contractures. His bilateral tendon reflexes were educed symmetrically. The Babinski sign was negative. Ophthalmic examination demonstrated esotropia and optic nerve atrophy. The serum CK level was 307–768 U/L. Leukodystrophy-related enzyme examination was normal. Blood and urine metabolism screens were normal. The karyotypic analysis was 46, XY. A brain MRI demonstrated frontal lobe micropolygyria, abnormal white-matter signals, and dysplasia of the brainstem and cerebellum (Fig. 2).

#### Patient 3

Patient 3 was a 1-year-6-month-old female born to a healthy mother by normal spontaneous vaginal delivery at full-term with a birth weight of 3.3 kg. She had one unaffected brother. Her parents were non-consanguineous Han from Beijing City (Pedigree shown in Supplementary Figure S1). The patient had feeding difficulties and decreased crying during infancy. She exhibited muscle weakness and hypotonia yet achieved head control at 4-months-old and the ability to sit at 1 and a half years of age. However, she could only sit unsupported for 2 minutes. She could not speak, but could understand easy instructions. Upon physical examination, she showed a prominent forehead, nystagmus, generalized muscle weakness, and marked hypotonia. Deep tendon reflexes were absent. Her Babinski sign was negative. An ophthalmic examination demonstrated retinal and choroidal pigment epithelium atrophy, as well as optic nerve atrophy (Fig. 1). Her serum CK level was 867 IU/l. Blood and urine metabolism screens were normal. A brain MRI showed frontal lobe micropolygyria, abnormal white-matter signals in the bilateral frontal region, dysplasia of the brainstem and cerebellum, and cerebellar cysts (Fig. 2).

### Mutation analysis by NGS

**Figure 3:**
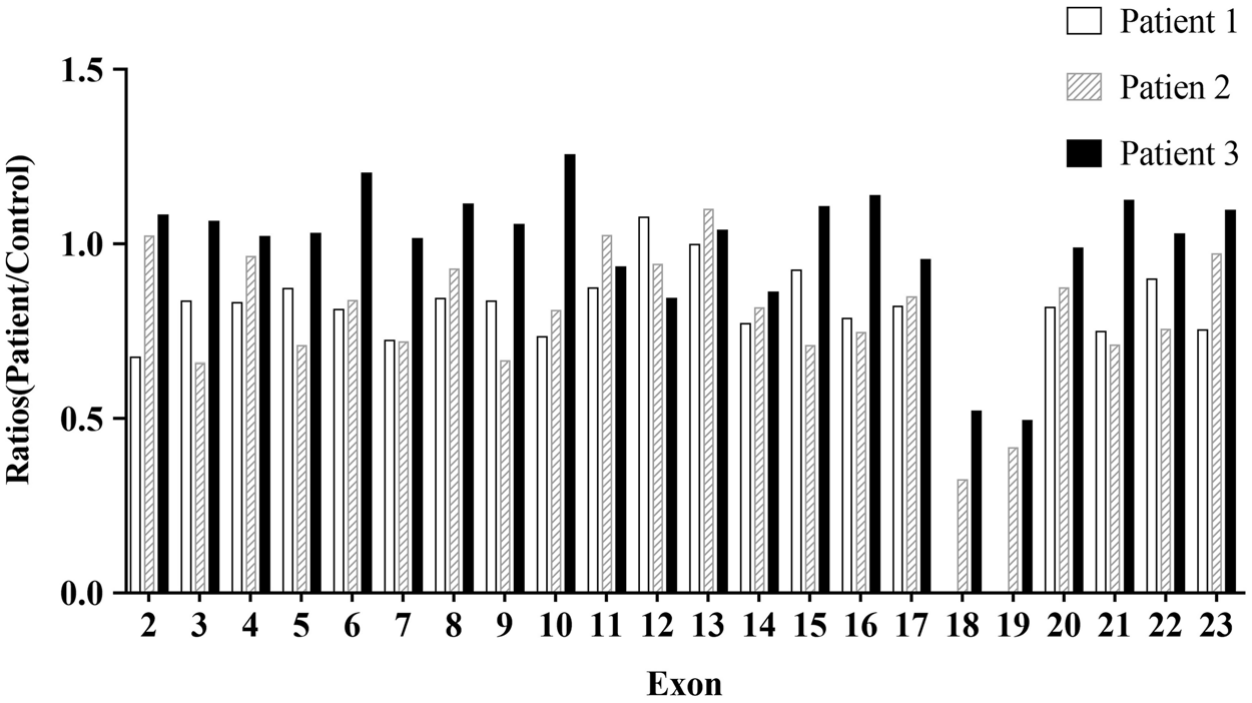
Patient/control ratios of the number each exon sequence reads. The patient/control ratios of all three patients were compared from exons 2 through 23 of *POMGNT1*, and performed near the level 1.0 on the vertical axis except the region of exons 18 and 19. The ratios of patient 1 were 0 in the region of exons 18 and 19, suggesting homozygous deletion of exons 18 and 19. The ratios of patient 2 and patient 3 were near 0.5 in the region of exons 18 and 19, suggesting heterozygous deletion of exons 18 and 19.

NGS revealed *POMGNT1* exons 18–19 deletion in all three patients, which was homozygous in patient 1 and heterozygous in patients 2 and 3. A summary of the number of patient and control reads of each exon, patient/control ratios of the number of sequence reads between patients, and their average control samples are listed (Table 1). The patient/control ratios of patient 1 were 0 in area of exons 18 and 19, which suggested homozygous deletion of exons 18 and 19. The patient/control ratios of patient 2 were 0.323317 in exon 18 and 0.415648 in exon 19. The patient/control ratios of patient 3 were 0.520472 in exon 18 and 0.49308 in exon 19. The ratios lower than 0.65 indicated patient 2 and patient 3 had heterozygous deletion of exons 18 and 19 (Fig. 3). Patient 2 had the compound heterozygous mutations of the CNV and a novel missense mutation c.296 T > C. Patient 3 had the compound heterozygous mutations of the CNV and a novel missense mutation c.794 G > C. Both missense mutations were located in the stem domain and predicted as pathogenic by using Polyphen-2, SIFT and Mutation Taster (Table 2).

**Table 1:** Summary of the number of each exon sequence reads from patient, the average number of each exon sequence reads from the 20 control samples, and patient/control ratio.

Exon	Region-begin	Region-end	Length (bp)	Number of aligned reads in this region		Ratios (Patient1/Control)	Number of aligned reads in this region		Ratios (Patient2/Control)	Number of aligned reads in this region		Ratios (Patient3/Control)
				Patient1	average		Patient2	average		Patient3	average	
2	chr1:46663364	chr1:46663503	139	82.994	122.9955	0.674773	159.644	156.3855	1.020837	5861	5423.05	1.080757
3	chr1:46662632	chr1:46662766	134	156.058	186.8846	0.83505	174.865	266.0134	0.657354	10918	10272.2	1.062869
4	chr1:46662393	chr1:46662531	138	125.182	150.7108	0.830611	179.453	186.4424	0.962512	9544	9357.45	1.019936
5	chr1:46661674	chr1:46661759	85	137.009	157.3698	0.870618	153.33	216.7542	0.707391	4649	4517.7	1.029063
6	chr1:46661473	chr1:46661606	133	92.266	113.7575	0.811076	132.981	158.9	0.836885	7131	5932.75	1.201972
7	chr1:46660506	chr1:46660643	137	117.127	162.2149	0.722048	146.924	204.6117	0.718063	6754	6666.25	1.013163
8	chr1:46660215	chr1:46660333	118	154.978	184.123	0.841709	233.18	251.7065	0.926396	8379	7526.55	1.113259
9	chr1:46659936	chr1:46660083	147	130.702	156.4079	0.835648	122.899	185.1609	0.663742	8196	7773.4	1.054365
10	chr1:46659517	chr1:46659607	90	124.36	169.7495	0.732609	202.153	250.2124	0.807925	8086	6449.8	1.253682
11	chr1:46659226	chr1:46659321	95	110.103	126.214	0.872352	181.078	176.9877	1.023111	2892	3098.7	0.933295
12	chr1:46658967	chr1:46659070	103	161.653	150.31	1.075464	196.21	208.8521	0.939469	4033	4785.45	0.842763
13	chr1:46658836	chr1:46658897	61	132.732	133.0002	0.997983	195.22	177.7368	1.098366	2280	2198	1.037307
14	chr1:46658562	chr1:46658640	78	141.616	183.8196	0.770407	210.889	258.5012	0.815814	4713	5477.85	0.860374
15	chr1:46658180	chr1:46658272	92	158.035	171.082	0.923738	194.035	274.6301	0.706532	6773	6126.15	1.105588
16	chr1:46657970	chr1:46658118	148	133.432	170.028	0.784765	186.556	250.459	0.744857	11522	10130.8	1.137324
17	chr1:46657760	chr1:46657905	145	162.464	198.1799	0.81978	237.271	280.0859	0.847136	9878	10360.2	0.953456
18	chr1:46656382	chr1:46656466	84	0	152.8705	0	71.752	221.9244	0.323317	2433	4674.6	0.520472
19	chr1:46656135	chr1:46656199	64	0	138.4967	0	84.412	203.0851	0.415648	1532	3107	0.49308
20	chr1:46655516	chr1:46655671	155	145.006	177.374	0.817516	225.92	258.8204	0.872883	11002	11144.8	0.987187
21	chr1:46655120	chr1:46655249	129	126.92	169.8529	0.747235	181.153	255.7618	0.708288	9401	8371.95	1.122916
22	chr1:46654904	chr1:46655039	135	131.699	146.6421	0.898098	160.808	213.266	0.754025	5875	5718.05	1.027448
23	chr1:46654381	chr1:46654662	281	115.252	153.1328	0.752628	205.46	211.9348	0.969449	17552	16027.4	1.095125

**Table 2:** Genotype of three MEB patients with *POMGNT1* (ENST00000371992, NM_001243766) mutations.

Patient No.	Nucleotide change	Amino acid change	Homo/Hete	Novel/Reported	Parental derivation	The Frequency of variant in GNOMAD and 1000 Genome	Pathogenicity prediction		
							PolyPhen2 (Score)	SIFT (Score)	Mutation Taster
1	g.6668–8257del	p.E514*	homozygous	Novel	Paternal/ Maternal	Not reported	Nonsense mutation	Nonsense mutation	Nonsense mutation
2	g.6668–8257del	p.E514*	heterozygous	Novel	Paternal	Not reported	Nonsense mutation	Nonsense mutation	Nonsense mutation
	c.296 T > C	p.L99P	heterozygous	Novel	Maternal	Not reported	Probably damaging(0.996)	Damaging(0.042)	Disease causing
3	c.794 G > C	p.R265P	heterozygous	Novel	Paternal	Not reported	Probably damaging(1.000)	Damaging(0.001)	Disease causing
	g.6668–8257del	p.E514*	heterozygous	Novel	Maternal	Not reported	Nonsense mutation	Nonsense mutation	Nonsense mutation

### Mutation analysis by long-range polymerase chain reaction (PCR) and Sanger sequencing

**Figure 4:**
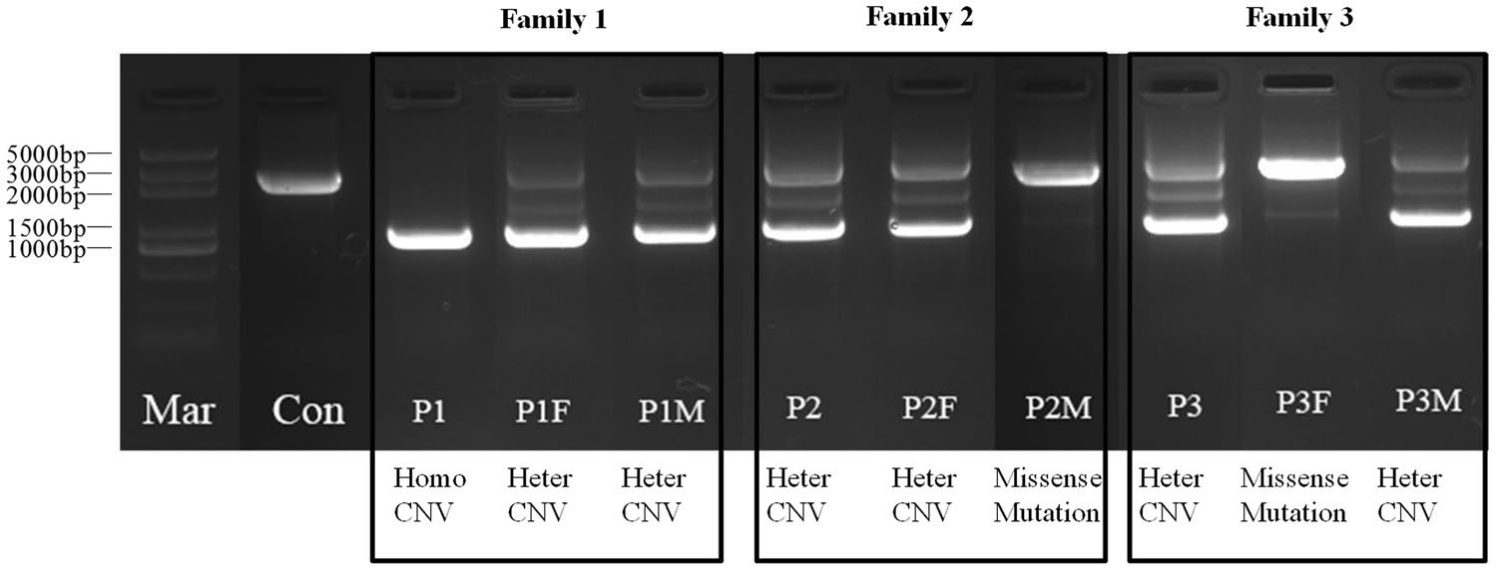
Electropherogram of PCR products of three MEB families. The normal control (Con) had only the predicted size 2476-bp band, the same as patient 2′s mother (P2M) and patient 3′s father (P3F) with only one missense mutation. Patient 1 (P1) with the homozygous (Homo) CNV had only one smaller size 886-bp band, while patient 1′s parents (P1F, P1M), patient 2 (P2), patient 2′s father (P2F), patient 3 (P3) and patient 3′s mother (P3M) with heterozygous (Heter) CNV had both the predicted size 2476-bp band and the smaller size 886-bp band. Full-length gels are presented in Supplementary Figure S2.

**Figure 5:**
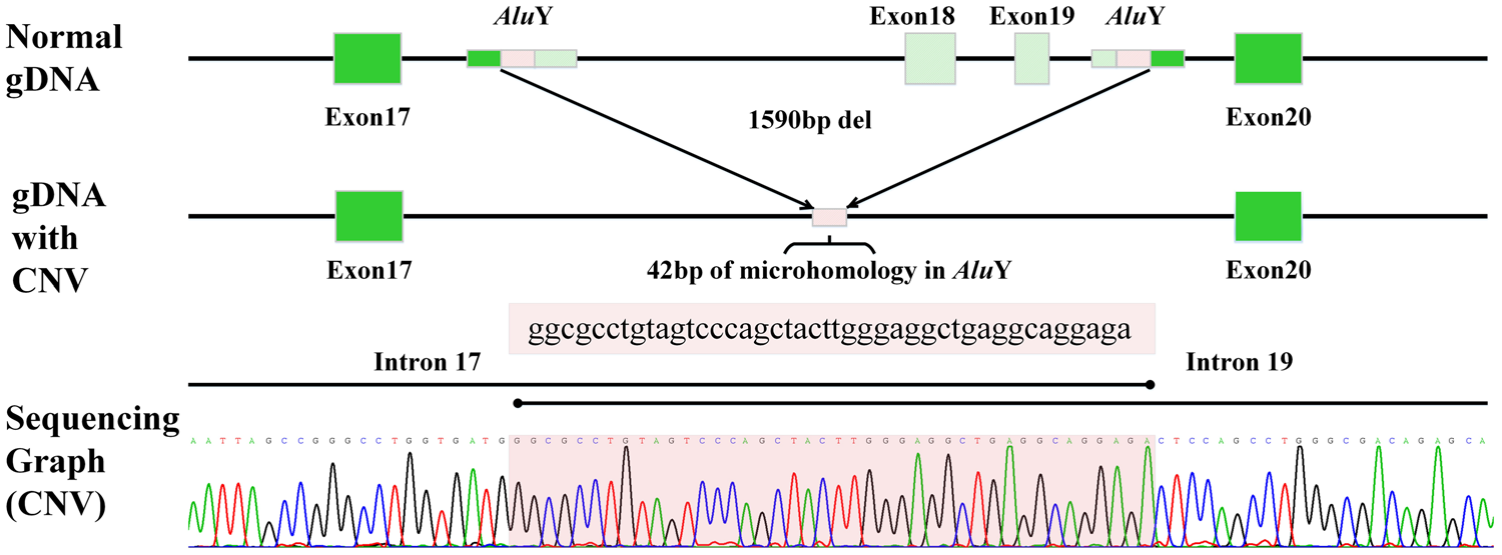
Length and breakpoints of the *POMGNT1* CNV. The 1590-bp CNV of *POMGNT1* included whole exons 18–19, as well as part of intron 17 and intron 19. The two breakpoints of the CNV localize to two *Alu*Y in intron 17 and intron 19. There are 42-bp of microhomology in two *Alu*Y resulting in *Alu-Alu* recombination.

An electropherogram of PCR products revealed that the normal control had only the predicted size 2476-bp band. Patient 1 with the homozygous CNV had only one smaller size 886-bp band, while patient 1′s parents, patient 2, patient 2′s father, patient 3, and patient 3′s mother with heterozygous CNV had both the predicted size 2476-bp band and smaller size 886-bp band (Fig. 4). Length and breakpoints of the CNV were also confirmed by long-range PCR and Sanger sequencing. Three patients had the same 1590-bp CNV, g.6668–8257del of *POMGNT1*, including whole exons 18–19, as well as part of intron 17 and intron 19. The two breakpoints of the CNV display 42-bp of microhomology and localize to two *Alu*Y elements found in the University of California, Santa Cruz (UCSC) genome browser (http://genome.ucsc.edu/) (Fig. 5).

### Prenatal diagnosis

All three DNA X chromosome markers (AR, DXS6797 and DXS6807) were informative in the family of Patient 2, with linkage analysis showing no maternal cell contamination in the amniocytes. We detected a carrier female fetus carrying only the paternal CNV(g.6668-8257del) without inheriting the maternal missense mutation (c.296 T > C). The girl was born uneventfully with no clinical MEB symptoms upon evaluation at 5 months old.

## Discussion

MEB is also called congenital muscular dystrophy-dystroglycanopathy with brain and eye anomalies (type A3; MDDGA3), an autosomal recessive disorder with characteristic brain and eye malformations, profound mental retardation, and congenital muscular dystrophy^11^. MDDGA3 includes the more severe WWS, which represents the most severe end of a phenotypic spectrum of similar disorders known as α-DGPs^5^. MEB had phenotypic similarities with WWS, and the distinctness of the MEB disease had been discussed and pointed to as having consistent muscle involvement and longer periods of survival^12, 13^. WWS is defined as prenatal or neonatal absence of motor development, severe structural brain abnormalities, including complete agyria or severe lissencephaly, marked hydrocephalus, severe cerebellar involvement, and complete or partial absence of the corpus callosum, as well as common eye abnormalities including congenital cataracts, microphthalmia, and buphthalmos^14, 15^. Death usually occurred before 1 year of age in WWS patients^5^.

In our study, three MEB patients from three unrelated families, including two males and one female, had similar manifestations and different clinical severity. Their similar manifestations included hypotonia at birth, mental retardation, structural brain defects, and ocular malformations. Moreover, patient 1 manifested more severely than patients 2 and 3. Patient 1 could not sit unsupported, and could neither speak nor communicate with others until his last visit when he was 8years and 5-months old. Patients 2 and 3 could sit unsupported and understand easy instructions. Patient 1 also had more severe brain and eye damage than patients 2 and 3, with manifestations that patients 2 and 3 did not have, such as microphthalmia, microcornea, right cataract, left severe myopia, septum pellucidum absence, corpus callosum dysplasia, and cerebellar cysts. Patient 1 was more likely to be diagnosed with WWS according to his severe clinical manifestations, but he had a longer survival span than 1 year, so he was also diagnosed with MEB.

Based on our previous experience^16^ and their specific phenotype, it was not hard to make a clinical diagnosis. However, it was challenging to make a genetic diagnosis. During the first NGS, no pathogenic mutation was found in patient 1, including all of the 18 known pathogenetic genes of α-DGPs. However, one *POMGNT1* mutation was found in patient 2 as well as patient 3, which indicated there was another undiscovered mutation. Considering that the sequencing quality was good enough, average sequencing reads were more than 100X, the targeted region was covered sufficiently, and no variant was omitted in the sequencing area, there were two possibilities. The first possibility was that the other undiscovered variant was a CNV. Otherwise, another variant possibly existed in the untranslated region (UTR) or in the introns. To detect variants in UTR and introns, another sequencing containing these areas needed to be done, so the existing NGS data were first used for detection of CNV. The number of each *POMGNT1* exon sequence reads from the three patients and the control samples were analyzed for CNV. Further long-range PCR and Sanger sequencing were conducted to confirm the CNV detected by NGS data analysis. The different band size and numbers of PCR products on the electropherogram revealed the CNV as homozygous or heterozygous, which is the same as the NGS data analysis result. These findings indicate that NGS is a clinically useful method of detecting *POMGNT1*-related CNV.

*POMGNT1* (ENST00000371992, NM_001243766) is divided into 23 exons and its coding region begins in exon 2. *POMGNT1* encodes a 748-amino acid type II transmembrane protein (Uniprot ID: Q8WZA1-2) with the region from Met1 to Arg37 constituting a cytoplasmic tail, Phe38 to Ile58 constituting a putative transmembrane domain, and the remaining lumenal region including a stem domain and a catalytic domain^1, 17–19^. The product of *POMGNT1*, protein O-mannose beta-1,2-N-acetylglucosaminyltransferase, participates in O-mannosyl glycan synthesis by transferring N-acetylglucosamine residues to O-linked mannose, which is essential for the proper functioning of α-DG^17, 20^. The CNV identified in *POMGNT1* is a novel, out-of-frame deletion immediately leading to a stop codon. Mutation Taster simulations predicted theproduction of a truncated protein lacking part of the catalytic domain, which would suggest that the CNV is pathogenic. Besides the same CNV identified in *POMGNT1*, two missense mutations c.296 T > C and c.794 G > C were detected in patients 2 and 3 respectively. The missense mutations c.296 T > C and c.794 G > C were both novel, but another mutation in the same position, c.794 G > A, had been reported in two MEB siblings from a non-Finnish Caucasian family^21^. Both novel mutations were located in the stem domain and predicted as pathogenic by Polyphen-2, SIFT, and Mutation Taster. Given the phenotype of patient 1 is more severe than patients 2 and 3, the genotype-phenotype correlation was that the patient with homozygous CNV was severe than patients with heterozygous CNV.

Most human repeat sequence is derived from transposable elements, including long interspersed elements (LINEs), short interspersed elements (SINEs), long-terminal repeat (LTR) retrotransposons, and DNA transposons. The first three types transpose through RNA intermediates and the last type transposes directly as DNA. *Alu* elements are the only active SINEs in the human genome, and they have a high mutagenic potential in humans^22^. At the molecular level, *Alu-Alu* recombination can lead to genomic deletion flanked by identical *Alu* repeats and it has been reported in some hereditary diseases^23^. To our knowledge, only two patients from North Africa and one patient from Italy with MEB caused by CNV in *POMGNT1* have been reported so far. The Italian patient had the deletion of exons 2–8 in *POMGNT1*, which was detected by quantitative PCR and multiplex ligation-dependent probe amplification (MLPA), with the breakpoints unconfirmed^24^. The two North African MEB patients had the deletion of exons 18–23 in *POMGNT1*, detected by quantitative PCR and array comparative genomic hybridization (aCGH). The breakpoints confirmed by long-range PCR were different in the CNV of the two North African MEB patients. They had different haplotypes, which demonstrated the occurrence of two independent events and supported a common mechanism involving unequal homologous recombination between highly similar *Alu*Y elements located in intron 17 and the 3′UTR region of *POMGNT1* ^25^. In our study, the Sanger sequencing of PCR products revealed the same breakpoints of CNV with 42-bp of microhomology, and showed that the two breakpoints of the CNV localized to two *Alu*Y elements in intron 17 and intron 19. Although exact rearrangement join-point locations were different between the CNV in our study and the CNV in the North African MEB patients, the centromeric breakpoints of these three CNV localized to the same *Alu*Y in intron 17. The finding supported that *Alu*Y in intron 17 may be a “hotspot” for large genomic rearrangements in *POMGNT1*, and the novel CNV g.6668–8257del discovered by NGS is likely to be caused by *Alu*-specific microhomology-mediated deletion. Moreover, haplotype analysis revealed that all three patients carrying this CNV had the same haplotype (Table 3), revealing that it may be a founder mutation from a single ancestor bearing the deletion chromosome.

**Table 3:** The results of haplotype analysis.

SNP ID	rs80107141	rs12737140	rs150578902	rs2292486	rs6659553
SNP of *POMGNT1* (NM_001243766)	c. − 11G > A	c.120 + 13 C > T	c.236 − 13 T > C	c.1111 − 23 C > T	c.1867A > G
Chromosome Position	1:46197832	1:46197689	1:46196862	1:46193238	1:46189486
Patient1	G(wild type)	C(wild type)	T(wild type)	T(homozygous)	G(homozygous)
Patient2	G(wild type)	C(wild type)	T(wild type)	T(heterozygous)	G(homozygous)
Patient3	G(wild type)	C(wild type)	T(wild type)	T(heterozygous)	G(homozygous)

To conclude, this study revealed how NGS can identify the CNV of MEB and other inherited diseases, and three novel mutations in *POMGNT1* were reported, which significantly expanded the mutation spectrum of MEB. The novel CNV g.6668–8257del discovered by NGS is likely to be caused by *Alu-Alu* recombination, or it results from a single ancestor bearing the deletion chromosome as a founder mutation. Furthermore, this study recognized that NGS plays an important role in genetic diagnosis, family genetics counseling, and prenatal diagnostic testing.

## Materials and Methods

### Patients

The research protocol was reviewed and approved by the Ethics Committee of the Peking University First Hospital (Beijing, China). All controls, patients, and/or their parents provided written informed consent to participate in the study and to grant permission to publish medical data. All methods were performed in accordance with the relevant guidelines and regulations. Clinical data were collected including motor development, mental development, pattern of muscle involvement, joint contracture, serum creatine kinase (CK) level, ocular malformation and brain involvement.

### NGS

Genomic DNA of the patients and their parents was extracted from peripheral blood lymphocytes. A custom panel was designed to sequence the 167 genes known to be related to inherited muscular disease (Genes list shown in Supplementary Table 1). First, a DNA library was prepared and amplified from genomic DNA. Then, the coding exons and partial introns of 167 genes were selected by the SureSelect target enrichment system kit (Agilent Technologies, Santa Clara, CA, USA). Sequencing was carried out on the Illumina NextSeq. 500 (Santiago, CA, USA). The results were transferred to a recognizable base sequence using bcl2fastq Conversion Software (Santiago, CA, USA). Clean reads were aligned on the human reference genome build hg19 using BWA^26^. Insertion-deletions (indels) and single-nucleotide polymorphisms (SNPs) were called using GATK software^27^ and functionally annotated by ANNOVAR^28^. Homozygous variants and heterozygous variants appearing twice or more in 50 normal controls were excluded. Prediction of pathogenicity for novel missense variants was performed using Polyphen-2 (http://genetics.bwh.harvard.edu/pph2/)^29^, SIFT (http://sift.jcvi.org/)^30^ and Mutation Taster (http://www.mutationtaster.org)^31^. Phylogenetic conservation analysis of missense mutations was also performed from several organisms.

### NGS data analysis for *POMGNT1* CNV detection

The NGS data were analyzed for detection of CNV by comparing the number of sequence reads between patient and control samples. First, the number of each exon sequence reads from patient and control was counted. For the purposes of this comparison, we used sequencing data from 20 control samples, including both males and females, without *POMGNT1* deletions (genomic sequencing was performed in these patients for diagnosis of other diseases). Data were obtained from samples using the same sequencing chip, sequenced on the same Illumina platform, and trimmed to the same read length as each patient sample. Next, the patient/control ratios were calculated by dividing the number of each exon sequence reads from patient by the average number of each exon sequence reads from the 20 control samples. Patient/control ratio lower than 0.65 indicates heterozygous deletions and ratios higher than 1.3 indicates duplications. Statistical analyses and graphs were obtained using GraphPad Prism7 (GraphPad Software, La Jolla, CA, USA).

### Long-range PCR and Sanger sequencing

Long-range PCR and Sanger sequencing were performed to verify and sequence the breakpoints of the CNV. Primers used for amplification of the deletion junction were designed using Primer 5.0 (Premier Biosoft, Palo Alto, CA, USA), including Primer F (5′-AGCTTGAGCCCAAGTGGCCTACACC-3′) and Primer R (5′-TGGGTCCAGGTGGTGAAGTCATCATC-3′). PCR conditions were as follows: 200 ng of genomic DNA was amplified in a 50ul reaction volume with 2ul of each Primer (10pmol), and 25ul 2 × Long Taq PCR MasterMix (KT203, TIANGEN Biotech, Beijing, China). PCR amplifications were performed in an Applied Biosystems Veriti Thermal Cycler (4375305, ThermoFisher Scientific, Waltham, MA, USA) for 35 cycles (30 s at 94 °C, 30 s at 60 °C and 180 s at 72 °C). PCR products were analyzed by electrophoresis on 2% agarose gel before sequencing. Then, PCR products were purified and directly sequenced using an automatic sequencer (ABI 3730xl DNA Analyzer; ThermoFisher Scientific, Waltham, MA, USA). Sequences were analyzed using DNASTAR software (Madison, WI, USA).

### Haplotype analysis

Haplotype analysis was performed in patient 1 with homozygous CNV, as well as patients 2 and 3 with heterozygous CNV, to detect *POMGNT1* SNPs. All were screened by NGS and Sanger sequencing for the following known polymorphisms: c.−11G > A; c.120 + 13 C > T; c.236 − 13 T > C; c.1111 − 23 C > T; c.1867A > G.

### Prenatal diagnosis

Prenatal diagnosis was carried out in Patient 2′s mother during a subsequent pregnancy at a gestational age of 21 weeks. A 20 ml amniotic fluid sample was collected by amniocentesis under ultrasound guidance and divided into two parts; one part (12 ml) was used to extract DNA from the fetal free cells, and the rest (8 ml) was placed in culture. Before DNA sequencing, maternal cell contamination was excluded by PCR linkage analysis of polymorphic microsatellite markers. Three DNA markers linked to the X chromosome (AR, DXS6797 and DXS6807) were used for linkage analysis^32^. Fetal sex was determined by karyotype analysis and PCR amplification of the Homo sapiens sex-determining region Y (SRY, NM_003140). Amniotic fetal DNA was extracted using the Wizard Genomic DNA Purification Kit (Promega, USA) according to the manufacturer’s instructions. The result of the fetal mutation analysis was confirmed by PCR amplification of the DNA extracted from the cultured amniocytes.

## Electronic supplementary material

Supplementary information

Supplementary Figure S1

Supplementary Figure S2
