# Supplementary material for: Novel copy number variation of POMGNT1 associated with muscle-eye-brain disease detected by next-generation sequencing
Source: Sci Rep. 2017 Aug 1;7:7056. doi: 10.1038/s41598-017-07349-8 (PMC5539251; doi:10.1038/s41598-017-07349-8)
Supplement: Supplementary file 1 — Supplementary information [file 41598_2017_7349_MOESM1_ESM.doc]

**Title Page**

**Novel copy number variation of *POMGNT1* associated with muscle-eye-brain disease detected by next-generation sequencing**

Xiaona Fu^1^, Haipo Yang^1^, Hui Jiao^1^, Shuo Wang^1^, Aijie Liu^1^, Xiaoqing Li^2^, Jiangxi Xiao^3^, Yanling Yang^1^, Xiru Wu^1^, Hui Xiong^1^*

^1^Department of Pediatrics, Peking University First Hospital, Beijing, 100034, China

^2^Department of child ophthalmology, Peking University First Hospital, Beijing 100034, China

^3^Department of Radiology, Peking University First Hospital, Beijing 100034, China

*Corresponding author: Hui Xiong, MD, PhD; Department of Pediatrics, Peking University First Hospital, No.1 Xi'an Men Street, West District, Beijing 100034, P.R. China, Tel.: +86 10 83573238; fax: +86 10 66530532; e-mail: [xh_bjbj@163.com](mailto:xh_bjbj@163.com)

**Supplementary Figure Legends**

Supplementary Figure S1. Pedigree of three families.

The parents of family 1 were consanguineous. They had an unaffected daughter, patient 1, two spontaneous abortions, and a termination of pregnancy when the fetus was 24 weeks because of fetal hydrocephalus. The parents of family 2 were non-consanguineous. They had patient 2 and an unaffected daughter. The parents of family 3 were non-consanguineous. They had an unaffected son and patient 3.

Supplementary Figure S2. Full-length gels of figure 4.

The uncropped image was labeled as in the main text. The last four samples in second row and all samples in third and fourth rows are unrelated to this experiment.

**Supplementary Table 1. Genes list of the panel.**

| **Gene** | **Description** | **OMIM No.** |
| --- | --- | --- |
| *ABHD5* | Abhydrolase domain containing 5 | 604780 |
| *ACADVL* | Acyl-Coenzyme A dehydrogenase, very long chain | 609575 |
| *ACTA1* | Actin, alpha 1, skeletal muscle | 102610 |
| *ACVR1* | Activin A receptor, type 1 | 102576 |
| *AGK* | Acylglycerol kinase | 610345 |
| *AGL* | Amylo-1, 6-glucosidase, 4-alphaglucanotransferase | 610860 |
| *AGRN* | Agrin | 103320 |
| *ALG13* | ALG13, S. Cerevisiae, Homolog of | 300776 |
| *ALG14* | ALG14, S. Cerevisiae, Homolog of | 12866 |
| *ALG2* | ALG2, S. Cerevisiae, Homolog of | 607905 |
| *ANO5* | Anoctamin 5 | 608662 |
| *ATP2A1* | ATPase, Ca++ transporting, fast twitch 1 | 108730 |
| *ATP5A1* | ATP synthase, H+ transporting, mitochondrial F1 complex, alpha subunit 1 | 164360 |
| *B3GALNT2* | Beta-1,3-N-acetylgalactosaminyltransferase 2 | 610194 |
| *B3GNT1* | beta-1,3-N-acetylglucosaminyltransferase 1 | 605517 |
| *BAG3* | BCL2-associated athanogene 3 | 603883 |
| *BIN1* | Bridging integrator 1 | 601248 |
| *CACNA1A* | Calcium channel,voltage-dependent, P/Q type,  alpha-1A subunit | 601011 |
| *CACNA1S* | Calcium channel, voltage-dependent, L type,  alpha-1S subunit | 114208 |
| *CAPN3* | Calpain 3 | 114240 |
| *CAV3* | Caveolin 3 | 601253 |
| *CCDC78* | Coiled-coil domain-containing protein 78 | 614666 |
| *CFL2* | Cofilin 2 | 601443 |
| *CHAT* | Choline acetyltransferase | 118490 |
| *CHKB* | Choline kinase, beta | 612395 |
| *CHRNA1* | Cholinergic receptor, nicotinic, alpha polypeptide 1 | 100690 |
| *CHRNB1* | Cholinergic receptor, nicotinic, beta polypeptide 1 | 100710 |
| *CHRND* | Cholinergic receptor, nicotinic, delta polypeptide | 100720 |
| *CHRNE* | Cholinergic receptor, nicotinic, epsilon polypeptide | 100725 |
| *CHRNG* | Cholinergic receptor, nicotinic, gamma polypeptide | 100730 |
| *CHST14* | carbohydrate sulfotransferase 14 | 608429 |
| *CLCN1* | Chloride channel 1,skeletal muscle | 118425 |
| *CNTN1* | Contactin 1 | 600016 |
| *COL12A1* | Collagen, type XII, alpha-1 | 120320 |
| *COL6A1* | Collagen, type VI, alpha-1 | 120220 |
| *COL6A2* | Collagen, type VI, alpha-2 | 120240 |
| *COL6A3* | Collagen, type VI, alpha-3 | 120250 |
| *COLQ* | Collagenic tail subunit of acetylcholinesterase | 603033 |
| *COQ8A* | Coenzyme Q8A | 606980 |
| *CPT2* | Carnitine palmitoyl transferase II | 600650 |
| *CRYAB* | Crystallin, alpha-B | 123590 |
| *DAG1* | Dystrophin-associated glycoprotein 1 | 128239 |
| *DARS* | Aspartate--tRNA synthetase | 603084 |
| *DES* | Desmin | 125660 |
| *DMD* | Dystrophin | 300377 |
| *DMPK* | Dystrophia myotonica protein kinase | 605377 |
| *DNAJB6* | DNAJ/HSP-40 homolog, subfamily B, member 6 | 611332 |
| *DNM2* | Dynamin 2 | 602378 |
| *DOK7* | Downstream of tyrosine kinase 7 | 610285 |
| *DOLK* | Dolichol kinase | 610746 |
| *DPAGT1* | Dolichyl-phosphate N-acetylglucosamine phosphotransferase | 191350 |
| *DPM1* | Dolichyl-phosphate mannosyltransferase 1, catalytic subunit | 603503 |
| *DPM2* | Dolichyl-phosphate mannosyltransferase 2, regulatory subunit | 603564 |
| *DPM3* | Dolichyl-phosphate mannosyltransferase 3 | 605951 |
| *DYSF* | Dysferlin | 603009 |
| *ECEL1* | Endothelin-converting enzyme-like 1 | 605896 |
| *EMD* | Emerin | 300384 |
| *ENO3* | Enolase 3 | 131370 |
| *ETFA* | Electron transfer flavoprotein, alpha polypeptide | 608053 |
| *ETFB* | Electron transfer flavoprotein, beta polypeptide | 130410 |
| *ETFDH* | Electron transferring flavoprotein dehydrogenase | 231675 |
| *FHL1* | Four-and-a-half LIM domains 1 | 300163 |
| *FKBP14* | FK506-binding protein 14 | 614505 |
| *FKRP* | Fukutin-related protein | 606596 |
| *FKTN* | Fukutin | 607440 |
| *FLNC* | Filamin C, gamma | 102565 |
| *GAA* | Glucosidase, alpha, acid | 606800 |
| *GBE1* | glycogen branching enzyme | 607839 |
| *GFPT1* | Glutamine: fructose-6-phosphate amidotransaminase 1 | 138292 |
| *GMPPB* | GDP-mannose pyrophosphorylase B | 615320 |
| *GNE* | UDP-N-acetylglucosamine 2-epimerase/N-acetylmannosamine kinase | 603824 |
| *GYG1* | Glycogenin 1 | 603942 |
| *GYS1* | Glycogen synthase 1 | 138570 |
| *HACD1* | 3-Hydroxyacyl-CoA dehydratase 1 | 610467 |
| *HADHB* | Hydroxyacyl-CoA Dehydrogenase/3-Ketoacyl-CoA Thiolase/Enoyl-CoA Hydratase, Beta Subunit | 143450 |
| *HSPG2* | Heparan sulfate proteoglycan of basement membrane | 142461 |
| *ISCU* | Iron–sulfur cluster scaffold, E. coli, homolog of | 611911 |
| *ISPD* | Isoprenoid synthase domain-containing protein | 614631 |
| *ITGA7* | Integrin, alpha-7 | 600536 |
| *ITGA9* | Integrin, alpha-9 | 603963 |
| *KBTBD13* | Kelch repeat-and BTB/POZ domain-containing protein 13 | 613727 |
| *KCNA1* | Potassium channel, voltage-gated, shaker-related subfamily, member 1 | 176260 |
| *KCNE3* | Potassium channel, voltage-gated, Isk-related subfamily, member 3 | 604433 |
| *KLHL40* | Kelch-like 40 | 615340 |
| *KLHL41* | Kelch-like 41 | 607701 |
| *KLHL9* | Kelch-like 9 | 611201 |
| *LAMA2* | Laminin, alpha-2 | 156225 |
| *LAMB2* | Laminin, beta-2 | 150325 |
| *LAMP2* | Lysosomal-associated membrane protein 2 | 309060 |
| *LARGE* | Acetylglucosaminyltransferase-like protein | 603590 |
| *LDB3* | LIM domain binding -3 | 605906 |
| *LDHA* | Lactate dehydrogenase A | 150000 |
| *LMNA* | Lamin A/C | 150330 |
| *LPIN1* | Lipin 1 | 605518 |
| *LRP4* | Low-density lipoprotein receptor-related protein 4 | 604270 |
| *MAMLD1* | Mastermind-like domain-containing protein 1 | 300120 |
| *MATR3* | Matrin 3 | 164015 |
| *MEGF10* | multiple EGF-like domains 10 | 612453 |
| *MSTN* | Myostatin | 601788 |
| *MTM1* | Myotubularin | 300415 |
| *MTMR14* | Myotubularin-related protein 14 | 611089 |
| *MUSK* | Muscle, skeletal, receptor tyrosine kinase | 601296 |
| *MYBPC1* | Myosin-binding protein C, slow-type | 160794 |
| *MYBPC3* | Myosin-binding protein C, cardiac | 600958 |
| *MYF6* | Myogenic factor 6 | 159991 |
| *MYH14* | Myosin, heavy chain 14, nonmuscle | 608568 |
| *MYH2* | Myosin, heavy chain 2, skeletal muscle, adult | 160740 |
| *MYH7* | Myosin, heavy chain 7, cardiac muscle, beta | 160760 |
| *MYOT* | Myotilin | 604103 |
| *NDUFB3* | NADH-ubiquinone oxidoreductase 1 beta subcomplex, 3 | 603839 |
| *NEB* | Nebulin | 161650 |
| *PABPN1* | Polyadenylate-binding protein, nuclear, 1 | 602279 |
| *PFKM* | Phosphofructokinase, muscle-type | 610681 |
| *PGAM2* | Phosphoglycerate mutase 2 | 612931 |
| *PGK1* | Phosphoglycerate kinase 1 | 311800 |
| *PGM1* | Phosphoglucomutase 1 | 171900 |
| *PHKA1* | Phosphorylase kinase, muscle, alpha-1 submit | 311870 |
| *PIEZO2* | Piezo-type mechanosensitive ion channel component 2 | 613629 |
| *PLEC* | Plectin | 601282 |
| *PLOD1* | Procollagen-lysine, 2-oxoglutarate 5-dioxygenase 1 | 153454 |
| *PLOD2* | Procollagen-lysine, 2-oxoglutarate 5-dioxygenase 2 | 601865 |
| *PLOD3* | Procollagen-lysine, 2-oxoglutarate 5-dioxygenase 3 | 603066 |
| *PNPLA2* | patatin-like phospholipase domain-containing protein 2 | 609059 |
| *POLG2* | Polymerase, DNA, gamma-2 | 604983 |
| *POMGNT1* | Protein O-mannose beta-1,2-N-acetylglucosaminyltransferase | 606822 |
| *POMGNT2* | Protein O-mannose beta-1,4-N-acetylglucosaminyltransferase 2 | 614828 |
| *POMK* | Protein-O-mannose kinase | 615247 |
| *POMT1* | Protein O-mannosyltransferase 1 | 607423 |
| *POMT2* | Protein O-mannosyl transferase 2 | 607439 |
| *PRKAG2* | Protein kinase, AMP-activated, noncatalytic, gamma-2 | 602743 |
| *PTRF* | RNA Polymerase I and transcript release factor | 603198 |
| *PUS1* | Pseudouridine synthase 1 | 608109 |
| *PYGM* | Glycogen phosphorylase, muscle | 608455 |
| *RAPSN* | Receptor-Associated Protein Of The Synapse, 43-KD | 601592 |
| *RBCK1* | RanBP-type and C3HC4- type zinc finger containing 1 | 610924 |
| *RYR1* | Ryanodine receptor 1 | 180901 |
| *SCN4A* | Sodium channel, voltage-gated, type IV, alpha subunit | 603967 |
| *SELENON* | Selenoprotein N | 606210 |
| *SGCA* | Sarcoglycan, alpha | 600119 |
| *SGCB* | Sarcoglycan, beta | 600900 |
| *SGCD* | Sarcoglycan, delta | 601411 |
| *SGCG* | Sarcoglycan, gamma | 608896 |
| *SGCE* | Sarcoglycan, epsilon | 604149 |
| *SIL1* | SIL1, S. Cerevisiae, Homolog of | 608005 |
| *SLC16A2* | Solute carrier family 16 (monocarboxylic acid transporter), member 2 | 300095 |
| *SLC22A5* | Solute carrier family 22 (organic cation transporter), member 5 | 603377 |
| *SLC25A20* | Solute carrier family 25(carnitine/acylcarnitine translocase), member 20 | 613698 |
| *SLC28A2* | Solute carrier family 28(sodium-coupled nucleoside transporter), member 2 | 606208 |
| *SMCHD1* | Structural maintenance of chromosomes flexible hinge domain-containing protein 1 | 614982 |
| *SYNE1* | Spectrin repeat-containing nuclear envelope protein 1 | 608441 |
| *SYNE2* | Spectrin repeat-containing nuclear envelope protein 2 | 608442 |
| *TCAP* | titin-cap | 604488 |
| *TIA1* | Cytotoxic granule-associated RNA-binding protein | 603518 |
| *TK2* | Thymidine kinase, mitochondrial | 188250 |
| *TMEM43* | Transmembrane protein 43 | 612048 |
| *TMEM5* | Transmembrane protein 5 | 605862 |
| *TNNT1* | Troponin T1, skeletal, slow | 191041 |
| *TNPO3* | Transportin 3 | 610032 |
| *TOR1AIP1* | Torsin A-interacting protein 1 | 614512 |
| *TPM2* | Tropomyosin 2 | 190990 |
| *TPM3* | Tropomyosin 3 | 191030 |
| *TRAPPC11* | Trafficking protein particle complex, subunit 11 | 614138 |
| *TRIM32* | Tripartite motif-containing protein 32 | 602290 |
| *TTN* | Titin | 188840 |
| *VCP* | Valosin-containing protein | 601023 |
| *YARS2* | Tyrosine--tRNA synthetase 2 | 610957 |
| *ZNF9* | Zinc finger protein 9 | 116955 |
