# Supplementary figures and images for: Novel copy number variation of POMGNT1 associated with muscle-eye-brain disease detected by next-generation sequencing
Source: Sci Rep. 2017 Aug 1;7:7056. doi: 10.1038/s41598-017-07349-8 (PMC5539251; doi:10.1038/s41598-017-07349-8)

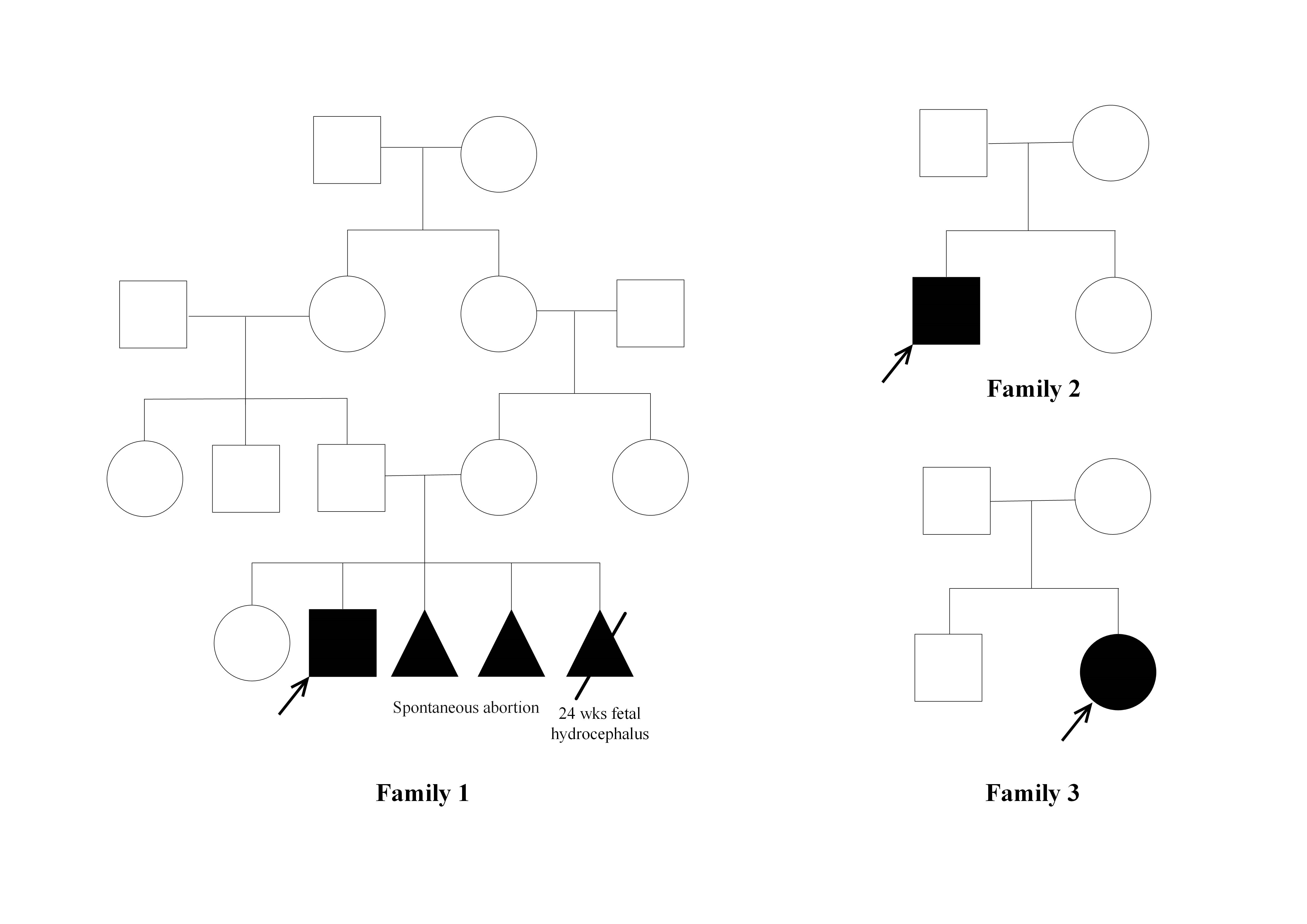

Supplement: Supplementary file 2 — Supplementary Figure S1 [file 41598_2017_7349_MOESM2_ESM.tif]

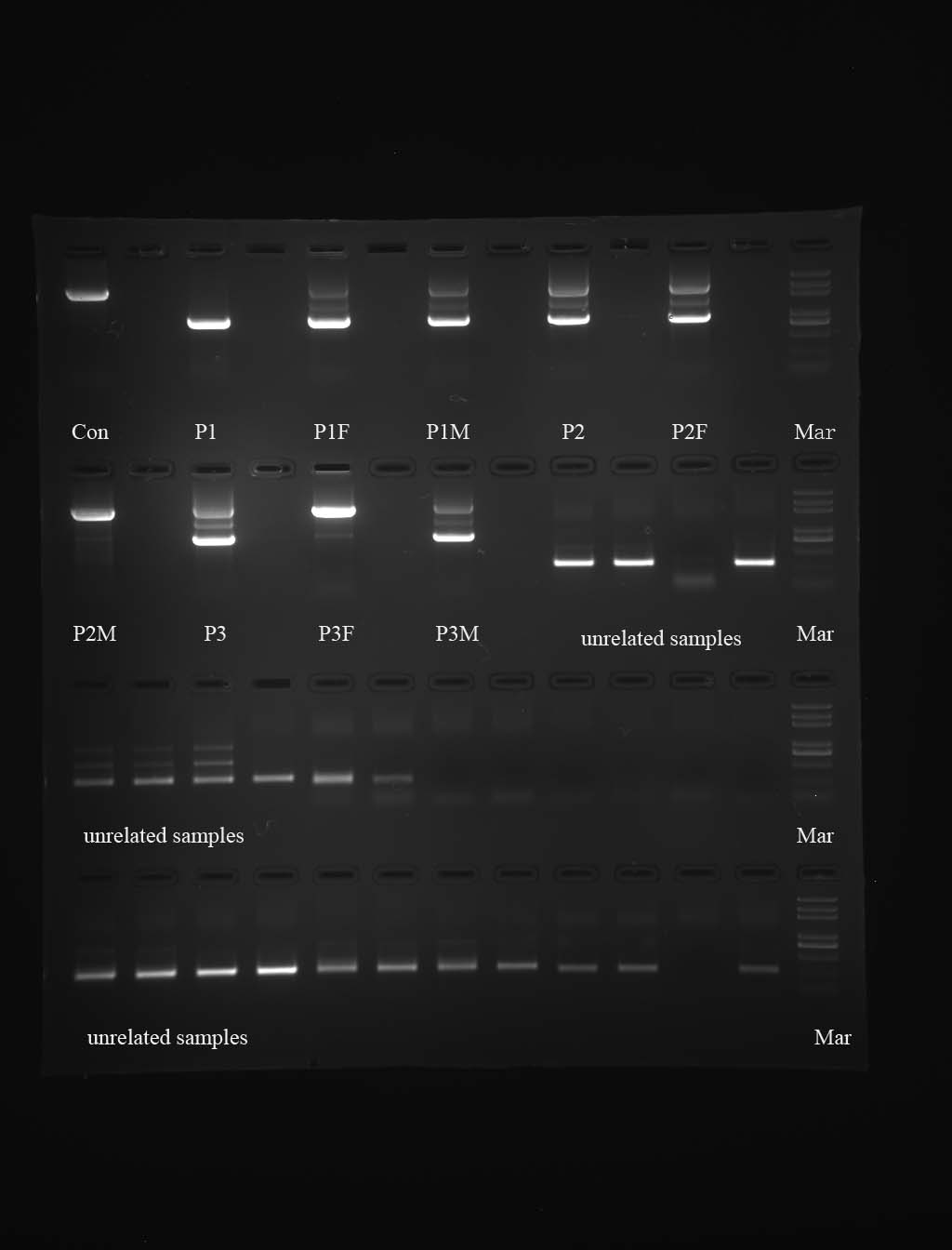

Supplement: Supplementary file 3 — Supplementary Figure S2 [file 41598_2017_7349_MOESM3_ESM.tif]
